# Effectiveness of Cleaning Methods for Resilient Denture Liners: A Three‐Period Randomized Crossover Trial

**DOI:** 10.1002/cre2.70243

**Published:** 2025-10-18

**Authors:** Rihoko Takeuchi, Shin Miyamae, Nana Sonobe, Humika Hattori, Yuei Morinaga, Hisato Hotta, Yoshiaki Hasegawa, Suguru Kimoto

**Affiliations:** ^1^ Department of Gerodontology and Home Care Dentistry, School of Dentistry Aichi Gakuin University Nagoya Japan; ^2^ Department of Oral Pathology/Forensic Odontology, School of Dentistry Aichi Gakuin University Nagoya Japan; ^3^ Department of Microbiology, School of Dentistry Aichi Gakuin University Nagoya Japan

**Keywords:** cleaning method, colony‐forming unit, complete denture, resilient denture liner

## Abstract

**Objective:**

Several studies have demonstrated that insufficient denture hygiene constitutes a significant risk factor for aspiration pneumonia. While various cleaning protocols have been proposed for conventional hard denture base materials, there remains a notable paucity of research specifically addressing effective cleaning strategies for resilient denture liners (RDLs). This study aimed to evaluate the efficacy of three denture cleaning methods—mechanical, chemical, and dual (combined mechanical and chemical)—for removing microbial biofilms from different denture‐liner materials using a randomized crossover clinical trial.

**Materials and Methods:**

This three‐period randomized crossover clinical trial included edentulous patients wearing maxillary complete dentures embedded with specimens of a hard‐resin liner and silicone‐based and acrylic‐based RDLs. All liners were fabricated directly in the patient's mouth. Participants performed the three cleaning interventions for 2 weeks each. Swabs from the relined denture surfaces were cultured in phosphate‐buffered saline, and the colony‐forming units (CFUs) were counted. The influence of each cleaning method on the CFU count was determined.

**Results:**

The dual‐cleaning method demonstrated a significant reduction in CFU count compared to mechanical cleaning, but only for the acrylic‐based RDL. No significant differences were observed between the cleaning methods for the silicone‐based RDL and hard‐resin liner. The CFU count was the highest for silicone‐based RDLs, followed by that for acrylic‐based RDLs, and hard‐resin liners.

**Conclusion:**

The dual method comprising mechanical and chemical cleaning was the most effective cleaning strategy for relined dentures, although the advantage was limited to acrylic‐based RDL.

**Trial Registration:**

This study was registered in the UMIN Clinical Trials Registry (UMIN000056738).

## 1 Introduction

Aspiration pneumonia is the sixth leading cause of death among older adults in Japan (IPSS 2025), underscoring its critical importance in geriatric healthcare. Older adults are at an increased risk of aspiration pneumonia owing to an age‐related decline in swallowing and immune functions (Ebihara et al. 2016). Yagi et al. (2016) reported that the mortality due to pneumonia is significantly higher in older adults than in other age groups, highlighting the importance of prevention and early intervention for aspiration pneumonia. Effective preventive measures include swallowing rehabilitation, nutritional management, and proper oral care (Langmore et al. 2002).

Denture cleaning is a crucial component of oral care for older adults. Several studies have shown that inadequate denture cleaning is a significant risk factor for aspiration pneumonia. A systematic review reported that inadequate denture cleaning leads to the accumulation of pathogens that, when aspirated, can cause pneumonia (Khadka et al. 2020). In addition, studies have shown that older adults who clean their dentures infrequently have a significantly higher risk of pneumonia (Kusama et al. 2019). Furthermore, people who wear dentures while sleeping often have poor denture hygiene and are 2.3 times more likely to develop pneumonia than those who remove their dentures while sleeping (Compagnoni et al. 2007; Iinuma et al. 2015). These findings highlight the critical role of keeping dentures clean in protecting the health of older adults and preventing aspiration pneumonia.

Clinical studies on denture cleaning methods have provided valuable insights into improving oral hygiene among denture wearers and reducing the risk of associated diseases. Baba et al. (2018) demonstrated that combining mechanical and chemical cleaning methods enhanced denture cleanliness and increased patient satisfaction compared to those using a single cleaning method. Similarly, a randomized crossover trial by Duyck et al. (2016) reported that the use of cleansing tablets during overnight storage, in addition to mechanical cleaning of dentures, reduces the total bacterial count on acrylic removable dentures more effectively than storage in water alone, with the effect being more pronounced when combined with ultrasonic cleaning rather than brushing. Lim et al. (2024) compared ultrasonic home‐denture cleaning with conventional cleaning methods, clinically validating the effectiveness of ultrasonic cleaning and demonstrating its utility. These clinical studies underscore the importance of selecting cleaning methods tailored to individual patients for maintaining denture cleanliness and preventing diseases.

However, to the best of our knowledge, these studies have mainly focused on dentures fabricated using conventional denture‐base materials and not on those with resilient denture liners (RDLs). Existing research on RDLs and cleaning methods has primarily emphasized the degradation of liners (Oliveira et al. 2006; Rodrigues Garcia et al. 2003; de Luna Malheiros Segundo et al. 2014), and no clinical study with high‐quality evidence has directly compared the effectiveness of different cleaning methods for these materials. Considering that RDLs are often applied to complete dentures to alleviate pain (Furokawa et al. 2020; Kimoto et al. 2007) and improve patient satisfaction and masticatory function (Furuya et al. 2022; 2021; Kimoto et al. 2014), establishing effective cleaning methods for soft denture liners is crucial. Therefore, we designed a randomized crossover clinical trial to evaluate effective cleaning methods for removing microbial biofilms formed on RDLs. The null hypothesis of this study is that there is no difference in the colony‐forming units (CFU) sampled from dentures cleaned using the mechanical cleaning method (mechanical method) with a denture brush, the chemical cleaning method with a denture cleanser (chemical method), and the dual cleaning method using both mechanical cleaning and a denture cleanser (Dual method).

## 2 Materials and Methods

### 2.1 Study Design

This three‐period crossover randomized clinical trial adhered to the principles of the Declaration of Helsinki and CONSORT guidelines. The trial was registered at the UMIN Clinical Trials Registry (UMIN000056738).

### 2.2 Participants and Ethical Approval

Participants were recruited from Aichi Gakuin University Dental Hospital from July 28, 2023 to February 28, 2025. Each participant received oral and written information regarding the study and provided informed consent. The study protocol was approved by the Ethics Committee of the School of Dentistry, Aichi Gakuin University (approval no. 670).

#### 2.2.1 Inclusion Criterion

The inclusion criteria were as follows: (1) Completely edentulous or maxillary completely edentulous and (2) willing to undergo a new complete denture treatment.

#### 2.2.2 Exclusion Criteria

The exclusion criteria were as follows: (1) Presence of severe systemic illness that would hinder participation in the study, (2) inability to understand and respond to the questionnaires, and (3) wearing complete dentures with a metal base.

### 2.3 Randomization, Allocation Concealment, and Sequence Generation

The six possible permutations of the three interventions were assigned numbers 1–6. Allocation numbers were subsequently generated using the “RAND” function in Excel (Microsoft Japan Co. Ltd., Tokyo, Japan). A randomization table was prepared, and participants were randomly assigned to one of the six sequence groups according to the table. Blinding of the participants was not feasible because they were instructed on cleaning their dentures before each period.

### 2.4 Specimen Preparation

Three cylindrical cavities (diameter, 5 mm and depth, 1 mm) were prepared on the palatal part of the intaglio surface of the maxillary complete denture base. One of the following mixed relining materials—acrylic‐based RDL (Biosoft Liner, Nissin), silicone‐based RDL (Tokuyama Sofreliner MS, Tokuyama), or hard resin liner (Tokuyama Rebase III, Tokuyama)—was filled using in each of the three cavities, and the denture was inserted in the patient's mouth in the proper position. The material was allowed to polymerize under occlusal pressure to ensure that the embedded specimens properly contacted the oral mucosa. After the polymerization, excess relining material was removed. The relining materials in the cavities were replaced at the beginning of each intervention to ensure that no colonies were present on the relined specimens.

### 2.5 Intervention

Participants followed a randomized cleaning schedule for 6 weeks, and based on the allocation table, switched cleaning methods every 2 weeks. At the end of each cleaning period, a swab test was performed on the filled cavity. The following instructions for each cleaning method were provided immediately before the corresponding intervention period.

1. Mechanical method
  Participants were instructed to rinse the dentures under running water and polish the entire surface with the flat side of the brush and the small areas with the pointed end of the brush. Brushing must be performed three times daily after meals. Additionally, participants must be instructed to store the dentures in water at bedtime.
2. Chemical method
  Participants were instructed about the chemical method: First, rinse the dentures under running water. Then, immediately after adding one tablet of the cleaning agent for RDLs (Tokuyama Rebase Cleaner, Tokuyama) to a denture case filled with lukewarm water, the dentures must be soaked overnight in the water. The dentures must be rinsed under running water before fitting. This must be performed once daily before going to bed.
3. Dual method

Participants were instructed to rinse the dentures under running water and polish the entire surface with the flat side of the brush and the small areas with the pointed end of the brush. Brushing must be performed three times daily after meals. Once daily, before going to bed, immediately after adding one tablet of the cleaning agent to a denture case filled with lukewarm water, the dentures must be soaked overnight in the water.

### 2.6 Outcome Measurement

#### 2.6.1 Colony‐Forming Units

##### 2.6.1.1 Sample Collection

After thoroughly removing saliva and debris from the surfaces of the specimens using running water, the specimens were air‐dried. Subsequently, a sterile cotton swab was used to collect samples by making careful wiping motions with consistent pressure several times. This procedure was performed for each specimen, and the swab was immediately placed in a 10 mL conical tube containing 1 mL of phosphate‐buffered saline. The samples were stored in a cool dark place until further analysis.

##### 2.6.1.2 Culture Method

Microbial culture was performed as previously described (Hayashi et al. 2022). The tubes containing the swabs and phosphate‐buffered saline solution were vortexed for 20 s. Next, the microbial suspension was transferred into a 2 mL microtube, and both undiluted and 10‐fold diluted solutions were prepared. Subsequently, 50 µL of each suspension was plated on Brain Heart Infusion agar medium and incubated under aerobic conditions at 37°C for 24 h.

##### 2.6.1.3 Counting Colony‐Forming Units

CFUs were counted as previously described (Hayashi et al. 2022). Briefly, after incubation, the formed colonies were counted and the total number of microbes collected from the specimen surfaces was calculated as CFU/mL. This method was used to quantitatively evaluate the microorganisms that adhered to each specimen.

### 2.7 Statistical Analyses

#### 2.7.1 Sample Size Calculation

In a previous study (de Almeida et al. 2020), the mean ± standard deviation CFU count in the pre‐ and post‐intervention groups was 6 ± 16 × 10^4^ CFU/mL and 1 ± 4 × 10^4^ CFU/mL, respectively. Based on these data, the required sample size to achieve 80% power with an alpha level of 5% was 58. The sample size was determined using G*Power version 3.1.9.4.

#### 2.7.2 Interim Analysis

An interim analysis was pre‐planned and conducted when approximately 30% of the target sample size completed the intervention. The primary objective of the interim analysis was to evaluate the effectiveness of the intervention on CFU counts and to determine whether the study should be continued, modified, or terminated. All statistical analyses were performed using G*Power version 3.1.9.4.

#### 2.7.3 Final Analysis

The intention‐to‐treat principle was applied to address any missing data after randomization. Missing values were replaced with the median of the data obtained for each intervention.

The Kolmogorov–Smirnov test was used to assess the normality of the CFU data. As the results indicated a non‐normal distribution, non‐parametric statistical methods were used. CFU data were analyzed using the Friedman test, followed by the Wilcoxon signed‐rank test with Bonferroni correction for multiple comparisons.

In a randomized crossover clinical trial, an appropriate assessment of the carryover effect, in which an intervention from a previous treatment period influences the subsequent intervention, is crucial to ensure the reliability of the trial results. To address this, the CFU values across the three periods for each cleaning method were compared using the Kruskal–Wallis test.

Categorical variables were compared using the chi‐square test, and continuous variables with normal and non‐normal distributions were compared using one‐way analysis of variance and the Kruskal–Wallis test, respectively. All statistical analyses were performed using SPSS Statistics v.21 (IBM, Armonk, NY, USA), and statistical significance was set at *p* < 0.05.

## 3 Results

### 3.1 Patient Flow

**Figure 1:**
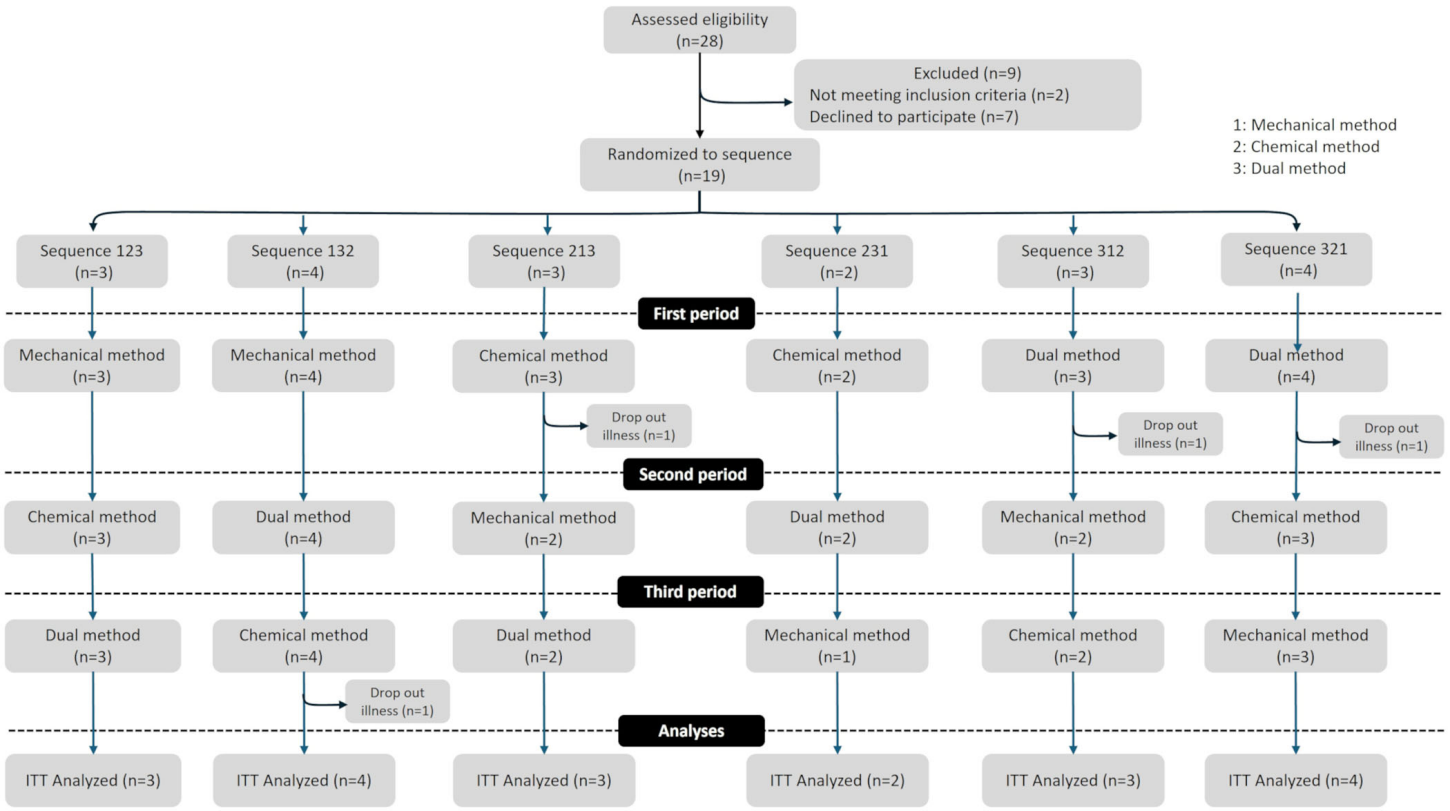
Patient flow. Sequence numbers 1, 2, and 3 represent the mechanical, chemical, and combined methods, respectively.

Figure 1 shows the study flowchart. A total of 28 participants were assessed for eligibility. Of these, nine were excluded (two did not meet the inclusion criteria and seven declined to participate). Finally, 19 participants were randomized into three groups corresponding to the six intervention sequences.

### 3.2 Colony‐Forming Units

#### 3.2.1 Effect of Interventions on CFU

**Figure 2:**
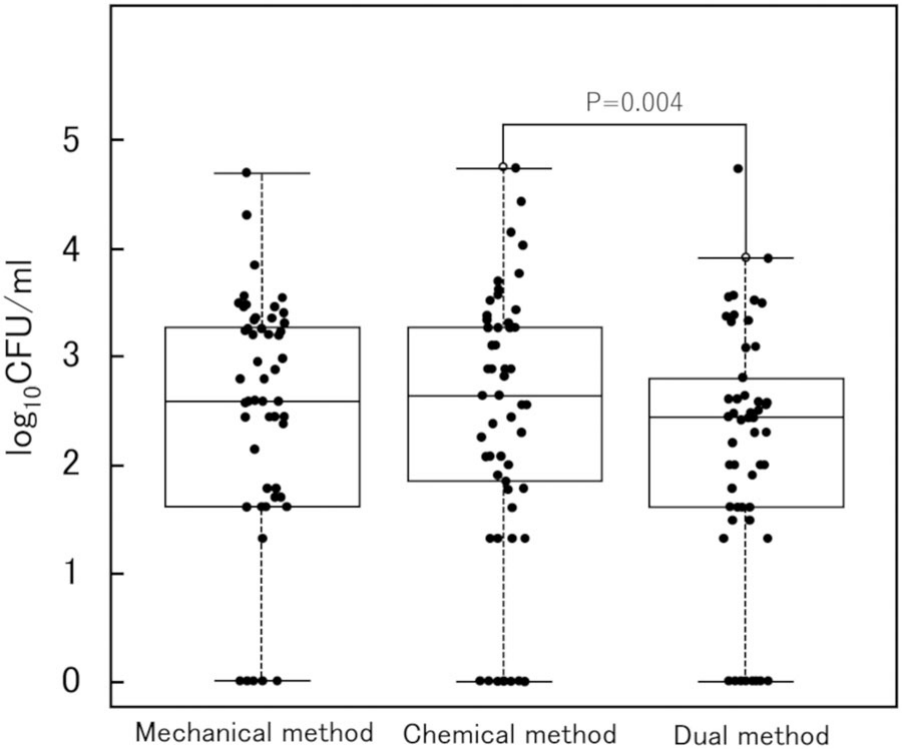
Effect of cleaning methods on CFU. The dual method was significantly more effective for reducing the CFU count than the mechanical method (*p* = 0.004). However, no statistically significant differences were observed among the other cleaning methods.

The box‐and‐whisker and jitter plots illustrate the CFU count distributions for the mechanical, chemical, and dual cleaning groups (Figure 2). The CFU counts for the three groups ranged from 0 to 49,800 (median, 390), from 0 to 55,400 (median, 440), and from 0 to 53,800 (median, 280), respectively. The Friedman test revealed a significant ranking of effectiveness among the three denture‐cleaning methods for reducing CFU (*p* = 0.05). Post hoc analysis demonstrated that the dual method was significantly more effective for reducing CFU than the chemical method (*p* = 0.004). However, no statistically significant differences were observed among the other cleaning methods.

**Figure 3:**
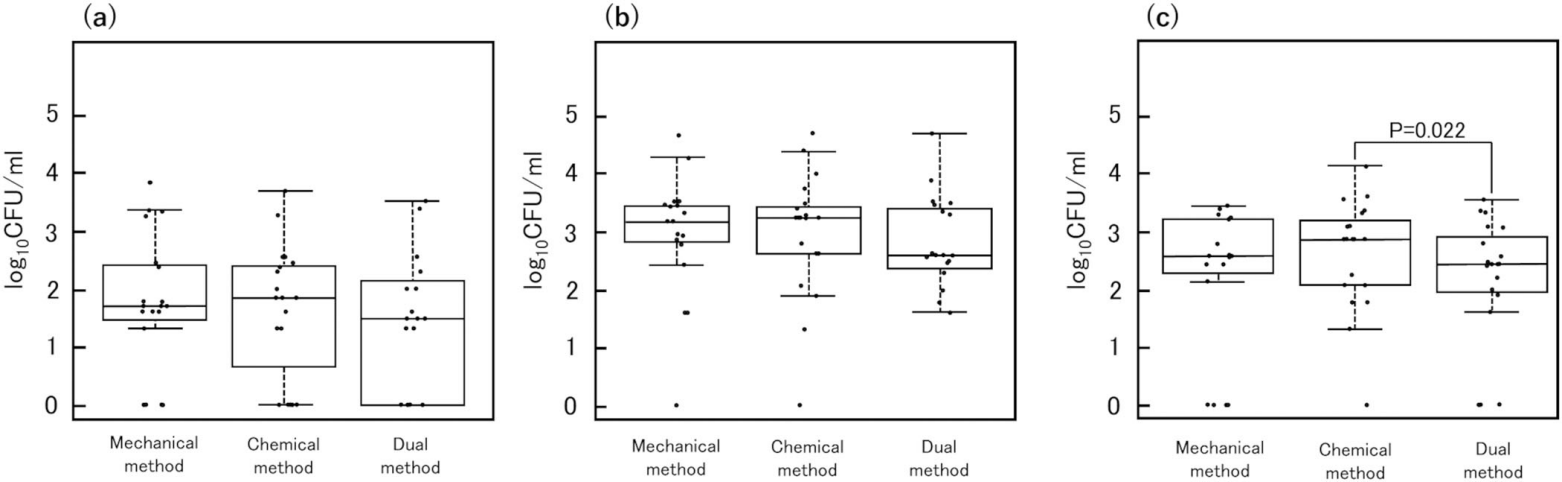
Effects of cleaning methods on CFU counts. (a) On hard‐resin liner. No statistically significant differences were observed between cleaning methods. (b) On silicone‐based RDL. No statistically significant differences were observed among the cleaning methods. (c) On acrylic‐based RDL. The dual method was significantly more effective at reducing CFU than the mechanical method (*p* = 0.022). CFU, colony‐forming unit; RDL, resilient denture liner.

Figure 3 illustrates the CFU reduction according to the material type using box‐and‐whisker and jitter plots. For the acrylic‐based RDL, the dual method was significantly more effective for reducing CFU than the chemical method (*p* = 0.022). However, for hard resin liners and silicone‐based RDLs, CFU reduction was not significantly different among the three methods.

#### 3.2.2 Effect of Relining Materials on CFU

**Figure 4:**
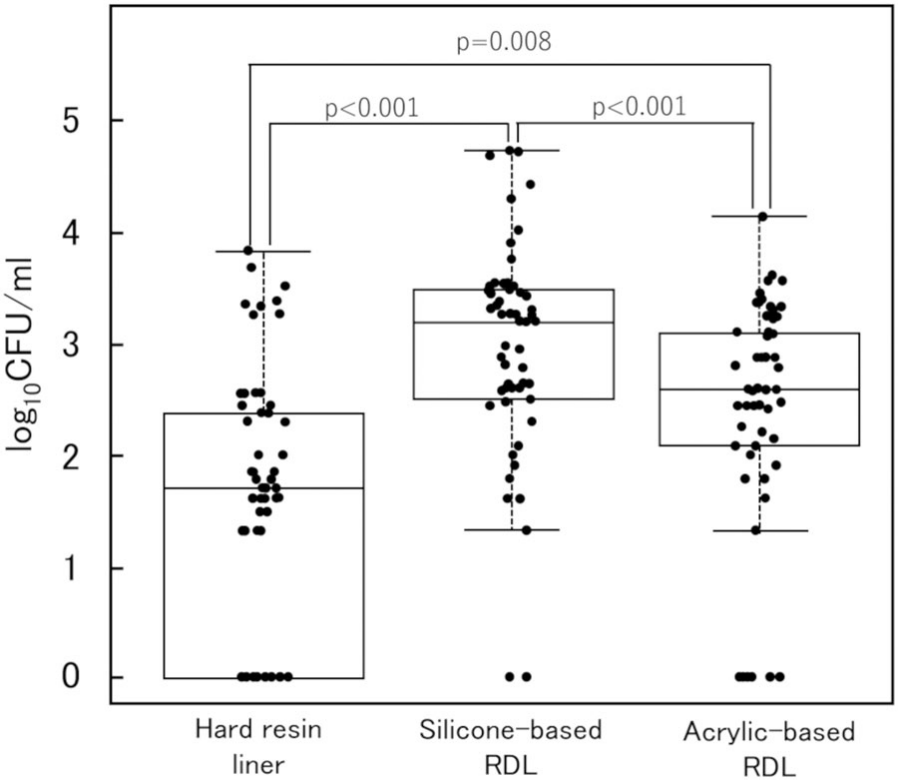
Effect of relining materials on CFU. Post hoc analysis demonstrated a statistically significant ranking, with CFU counts being the highest for the silicone‐based RDL, followed by the acrylic‐based RDL, and hard‐resin liner. CFU, colony‐forming unit; RDL, resilient denture liner.

The box‐and‐whisker and jitter plots illustrate the CFU count distributions for the hard‐resin liner, silicone‐based RDL, and acrylic‐based RDL (Figure 4). CFU counts in the three groups ranged from 0 to 6960 (median, 50), from 0 to 55,400 (median, 1600), and from 0 to 14,000 (median, 390), respectively. The Friedman test revealed that the reduction in CFU was significantly influenced by the type of relining material (*p* < 0.001). The silicone‐based RDL showed significantly higher CFU counts than the hard‐resin liner and acrylic‐based RDL (*p* < 0.001). The acrylic‐based RDL showed significantly higher CFU counts than the hard‐resin liner (*p* = 0.008). The CFU count was the highest for silicone‐based RDLs, followed by acrylic‐based RDL and hard‐resin liners.

#### 3.2.3 Carry Over Effect of CFU

The Kruskal–Wallis test revealed no significant differences in CFU counts for each cleaning method across the three periods, indicating the absence of a carry‐over effect.

### 3.3 Interim Analysis

An interim analysis was conducted after 19 participants had completed the study. CFU counts were not significantly different between the mechanical and chemical cleaning methods and between the mechanical and dual methods. The effect size (Cohen's *d*) between the mechanical and chemical methods and the mechanical and dual methods was 0.08 and 0.06, respectively, which was below the threshold of clinical significance. Based on these results, the study was terminated, as further recruitment of subjects would not enable the detection of clinically significant differences.

## 4 Discussion

This study aimed to identify effective cleaning methods for removing microbial biofilm formed on RDLs. Then, the dual method combining mechanical and chemical cleaning was the most effective cleaning method. However, this efficacy was observed only for acrylic‐based RDL. In an observational study, Nishi et al. (2012) reported that the use of a denture brush combined with the daily use of a denture cleanser was an effective cleaning method that significantly reduced the microbial load on complete dentures. Although limited to the efficacy for biofilm removal from specific materials, the results of our study are consistent with these findings.

In this study, no significant advantage of the dual method over other cleaning methods was observed for hard‐resin liners and silicone‐based RDLs. The low microbial growth on several specimens must be considered. Microorganisms begin to adhere to elastic denture liners as early as 14 h after exposure (Graham et al. 1991). However, Valentini et al. (2013) and Pereira‐Cenci et al. (2007) reported that microbial biofilms needed to mature after 21 days. De Almeida et al. (2020) started after 14 days to collect biofilm for biofilm maturation. Based on these reports, the early intervention immediately after relining likely resulted in insufficient biofilm maturation in our study. This premature intervention may have made observing differences in CFU levels between interventions difficult. In addition, swabbing a 5‐mm diameter specimen may have resulted in a limited number of microorganisms sampled. To provide robust evidence, future clinical trials should be conducted using protocols that address these issues.

The secondary finding of this study was that CFU values followed the order silicone‐based RDLs > acrylic‐based RDLs > hard denture liners. Extensive research has been conducted on microbial adhesion to RDLs, with detailed discussions of the underlying mechanisms. Silicone‐based RDLs have been widely reported to promote bacterial and fungal adhesion because of their hydrophobicity and surface roughness. Valentini et al. (2013) reported that the surface roughness of silicone‐based RDLs significantly influenced microbial adhesion, and biofilm formation increased with increasing surface roughness. Hahnel et al. (2012) demonstrated that hydrophobic surfaces of silicone‐based RDLs are particularly susceptible to initial microbial adhesion, highlighting the susceptibility of these materials to microbial colonization. Pereira‐Cenci et al. (2007) reported that biofilm maturation on silicone‐based RDLs can lead to microbial infiltration of surface voids, thereby reducing the effectiveness of cleaning methods. In contrast, regarding acrylic‐based RDLs, Chladek et al. (2014) suggested that water absorption facilitates bacterial and fungal penetration of the liner material, thereby promoting biofilm formation. Due to the characteristics of RDLs, we believe both types of RDLs might exhibit greater microbial adhesion than the hard‐resin liner. Furthermore, Pereira‐Cenci et al. (2007) reported that acrylic‐based RDLs exhibit hardening and surface degradation after prolonged use, which further increases microbial adhesion. Because the specimens were replaced biweekly in this study, the acrylic‐based RDLs did not undergo deterioration, which could have contributed to the higher levels of microbial adhesion observed on the silicone‐based RDLs. In addition, overnight storage conditions may influence denture geometry and liner properties (Lawson et al. 2024; Lim and Lee 2016). Our instructions specified overnight water storage; however, studies report that dry storage induces greater deformation/warpage than wet storage. Storage and cleansing media might also modify the hardness and sorption behavior of denture liners, potentially altering surface topography and microbial adhesion. Thus, future studies should investigate the differences in microbial adhesion between wet and dry storage conditions.

While this study focused on bacteria that grow under aerobic conditions, it is important to acknowledge that obligate anaerobic bacteria may also contribute to the microbial environment in the oral cavity. In particular, obligate anaerobes—especially gram‐negative, proteolytic species—tend to dominate dental biofilms under low‐oxygen conditions and are closely associated with periodontal infections (Larsen and Fiehn 2017). In this study, the bacteria analyzed mainly included aerobic and facultative anaerobic species grown under aerobic culture conditions. However, it is necessary to discuss the potential impact of the cleaning method on obligate anaerobic bacteria and their possible roles. The cleaning method evaluated may affect obligate anaerobic bacteria by altering the overall microbial environment or disrupting biofilm structures that harbor both aerobic and anaerobic organisms. Future studies should include anaerobic bacterial assessments to provide a more comprehensive evaluation of cleaning efficacy.

The interim analysis revealed no significant differences in the primary outcome between the mechanical method and the dual method, nor between the mechanical method and the chemical method. Moreover, the observed effect size was below the threshold for clinical significance, and projections indicated that a substantial increase in sample size would be required to justify continuation of the trial. These results implied the limitations of the current study protocol. Implementing protocols addressing factors such as increasing the number of microorganisms collected by expanding the specimen collection area and accounting for the maturation period of the microorganisms in future studies. This study is in a preliminary phase and is therefore regarded as a pilot study.

Although this study is positioned as a pilot study, it had several clinical implications: the dual cleaning method demonstrated efficacy for acrylic‐based RDL, highlighting the importance of implementing material‐specific cleaning protocols for denture liners. In addition, careful follow‐up is recommended when prostheses are relined with RDLs because these materials are highly susceptible to microbial adhesion.

## 5 Conclusion

Within the limitations of this study, the dual method comprising mechanical and chemical cleaning was the most effective cleaning strategy for relined dentures. However, its effectiveness was limited to acrylic‐based RDL. The dual method did not show any significant superiority over other methods for the hard‐resin liner or silicone‐based RDL. Notably, silicone‐based RDLs showed the highest CFU counts, followed by acrylic‐based RDLs and hard‐resin liners.

## Author Contributions

Rihoko Takeuchi contributed to the study design, data collection, and data analysis. Shin Miyamae contributed to the study design and data collection. Nana Sonobe provided advice on the study design and methodology. Hisato Hotta and Humika Hattori assisted in the preparation and editing of the English manuscript. Yuei Morinaga contributed to data interpretation and statistical analysis. Yoshiaki Hasegawa, as a professor of microbiology, supervised the bacterial culture procedures. Suguru Kimoto supervised the study overall and drafted the manuscript. All authors reviewed and approved the final version of the manuscript.

## Conflicts of Interest

The authors declare no conflicts of interest.

## Data Availability

The data that support the findings of this study are available from the corresponding author upon reasonable request.
